# PURα Promotes the Transcriptional Activation of *PCK2* in Esophageal Squamous Cell Carcinoma Cells

**DOI:** 10.3390/genes11111301

**Published:** 2020-10-31

**Authors:** Yan Sun, Jiajia Gao, Zongpan Jing, Yan Zhao, Yulin Sun, Xiaohang Zhao

**Affiliations:** State Key Laboratory of Molecular Oncology, National Cancer Center/National Clinical Research Center for Cancer/Cancer Hospital, Chinese Academy of Medical Sciences and Peking Union Medical College, Beijing 100021, China; sybil2018@student.pumc.edu.cn (Y.S.); gao19930111@foxmail.com (J.G.); jing19941@foxmail.com (Z.J.); zhaoyan1982@foxmail.com (Y.Z.); ylsun@cicams.ac.cn (Y.S.)

**Keywords:** PURα, PCK2, esophageal squamous cell carcinoma, transcriptional activation, metabolism

## Abstract

Esophageal squamous cell carcinoma (ESCC) is one of the most lethal gastrointestinal malignancies due to its characteristics of local invasion and distant metastasis. Purine element binding protein α (PURα) is a DNA and RNA binding protein, and recent studies have showed that abnormal expression of PURα is associated with the progression of some tumors, but its oncogenic function, especially in ESCC progression, has not been determined. Based on the bioinformatic analysis of RNA-seq and ChIP-seq data, we found that PURα affected metabolic pathways, including oxidative phosphorylation and fatty acid metabolism, and we observed that it has binding peaks in the promoter of mitochondrial phosphoenolpyruvate carboxykinase (PCK2). Meanwhile, PURα significantly increased the activity of the *PCK2* gene promoter by binding to the GGGAGGCGGA motif, as determined though luciferase assay and ChIP-PCR/qPCR. The results of Western blotting and qRT-PCR analysis showed that PURα overexpression enhances the protein and mRNA levels of PCK2 in KYSE510 cells, whereas PURα knockdown inhibits the protein and mRNA levels of PCK2 in KYSE170 cells. In addition, measurements of the oxygen consumption rate (OCR) and extracellular acidification rate (ECAR) indicated that PURα promoted the metabolism of ESCC cells. Taken together, our results help to elucidate the molecular mechanism by which PURα activates the transcription and expression of PCK2, which contributes to the development of a new therapeutic target for ESCC.

## 1. Introduction

Esophageal squamous cell carcinoma (ESCC) is one of the deadliest malignant tumors in the world. Although the management and treatment of patients with ESCC have been improved, the 5-year survival rate (~20–30%) of patients with ESCC is still low [1]. In recent years, large-scale genome sequencing and protein maps have provided novel insights into the diagnosis of early esophageal cancer, the prediction of susceptibility genes and the discovery of biomarkers that may be used in targeted therapy for ESCC [2]. An increasing number of researchers have mainly explored ESCC in terms of tumor immunity, lncRNAs, drug therapy and biomarkers [3,4,5,6].

The human purine-rich element binding protein (PUR) consists of the Pur-alpha (PURα) and Pur-beta (PURβ) and two forms of the Pur-gamma (PURγ) proteins, respectively [7]. Among these proteins, PURα contains 322 amino acids. PURα is a sequence-specific single-stranded DNA and RNA binding protein that participates in a variety of biological processes, including myoblast differentiation, hematopoietic and nervous system development, virus activation, tumorigenesis and drug resistance [8,9,10,11,12,13]. The protein sequence of PURα with three repeated nucleic acid binding domains is highly conserved from bacteria to humans [11,14] and plays an important role in the development of the mammalian hematopoiesis and the central nervous system. *PURA* homozygous knockout mice survive briefly, exhibit movement disorders and develop memory deficits after birth [15,16]. In addition, deletions or mutations in *PURA* have now been implicated in acute myeloid leukemia (AML), myelodysplastic syndrome (MDS) and *PURA* syndrome characterized by neonatal hypotonia, respiratory compromise, feeding difficulties, severe intellectual disability and epilepsy [17,18,19,20]. Moreover, abnormal expression of PURα is also correlated with proliferation and the anchorage-independent colony formation of ovarian or prostate cancer cells [12,21,22]. Recent elucidation of the abnormal expression of PURα in ESCC helps to provide a basic-research support for the tumor progression [23,24]. Similarly, our previous research observed high expression of PURα in ESCC tissue samples, suggesting that PURα plays an essential role in ESCC (unpublished data). However, the molecular pathways involved in the occurrence, development and treatment of ESCC are very complex [25], and little is known regarding the oncogenic function of PURα in ESCC. Consequently, we performed a chromatin immunoprecipitation sequencing (ChIP-seq) and RNA-seq analysis of ESCC cells to determine the global occupancy and underlying functions of PURα. Through the bioinformatic analysis of ChIP-seq and RNA-seq, we found that PURα was strongly associated with metabolic pathways and that mitochondrial phosphoenolpyruvate carboxykinase (*PCK2*) was an optimal candidate gene for PURα. Our results demonstrated the novel specific motifs and functions of PURα and elucidated the transcriptional regulation mechanism of PURα for the *PCK2* gene. The results of this study may serve to elucidate the molecular mechanism of ESCC and facilitate the diagnosis and treatment of ESCC.

## 2. Materials and Methods 

### 2.1. Cell Culture

The human ESCC cell lines KYSE510 and KYSE170 were generously provided by Dr Shimada (Hyogo College of Medicine, Hyogo, Japan). KYSE170-shNC and KYSE170-shPURα32/34 cells, stable PURα-knockdown cells, were constructed via infection with shPURα32/34 lentiviral particles (Fulengen, Guangzhou, China) and selection with 100 μM hygromycin B (Invitrogen, Carlsbad, CA, USA) for more than 2 weeks. The shRNA sequences are listed in Table 5. KYSE510-pCMV6 and KYSE510-PURα, stable PURα-overexpressing cells, were generated via transfection of the pCMV6 and pCMV6-PURα plasmid into KYSE510 cells, respectively, and selection with 400μg/mL G418 (Sigma-Aldrich, St. Louis, MO, USA) was for more than 2 weeks. The cells were cultured in RPMI 1640 medium (GE Healthcare Life Sciences, Pittsburgh, PA, USA) with 10% fetal bovine serum (FBS) (HyClone, Logan, UT, USA) in a humidified cell incubator (NAPCO, Winchester, VA, USA) with 5% CO_2_ at 37 °C.

### 2.2. RNA Extraction, Real-Time Quantitative PCR (qRT-PCR) and RNA Sequencing (RNA-seq)

Total RNA from cells was isolated and extracted with a TRIzol kit (Invitrogen, Carlsbad, CA, USA) and Direct-zolTM RNA MiniPrep kit (Zymo Research, Irvine, CA, USA). Reverse transcription synthesis of cDNA was performed using 2 μg RNA as template and a HiFiScript cDNA Synthesis kit (CWBIO, Beijing, China). Real-time quantitative RT-PCR was performed out using a TB Green^®^ Premix Ex Taq quantitative kit (Tli RNaseH Plus) (Takara, Kyoto, Japan) and QuantStudio quantitative 5 instrument (Applied Biosystems, Foster, CA, USA). The expression of mRNA for all genes was normalized to β-actin. The primer sequences are listed in Table 1, and the final raw data were analyzed by the 2^−∆∆Ct^ calculation method [26].

Total RNA from KYSE510-pCMV6 and KYSE510-PURα stable cells was isolated from 1 × 10^7^ cells and treated with RQ1 DNase (Promega, Wisconsin, WI, USA) to remove DNA. Polyadenylated mRNAs were purified and concentrated with oligo (dT)-conjugated magnetic beads (Invitrogen, Carlsbad, CA, USA) before directional RNA-seq library preparation. Then, the purified mRNAs were fragmented at 95°C followed by end repair and 5' adaptor ligation and the cleaved RNA fragments were reverse-transcribed to generate cDNAs using primers containing a 3' adaptor sequence and random hexamers. The cDNAs were purified and amplified and PCR products corresponding to 200-500 bp were purified, quantified and stored at −80 °C until used for sequencing was performed. 

For high-throughput sequencing with biological triplicates per group, the Illumina HiSeq X Ten (Illumina, San Diego, CA, USA) system was used to obtain 150-nt paired-end sequencing reads by ABLife Inc. (Wuhan, China). Briefly, the clean reads were generated after removing adaptor sequences and low-quality sequences. A FASTX-Toolkit (Version 0.0.13) software was used to obtain the filtered clean reads. Then, filtered reads were mapped to human genome hg38 using TopHat2 version 2.1.0 [27] with default settings and reads summarized by gene feature using htseq-count [28]. Expression levels of each gene were calculated by counting the number of sequenced tags mapped to the gene and normalized by mapped reads per kilo base of exon per million mapped reads (RPKM) based on a previous method [29]. Differentially expressed genes (DEGs) with a RPKM logFC ≥ 0.5 or ≤ −0.5 and *p* < 0.01 were analyzed using the edgeR package embedded in R software [30].

### 2.3. ChIP-seq and ChIP-PCR Assay

Transcriptome sequencing libraries were prepared using the ThruPLEX DNA-seq Kit (Rubicon Genomics, Ann Arbor, MI, USA) by following the manufacturer’s procedure. Whole cell extracts of KYSE510-PURα cells were prepared from formaldehyde fixed cells resuspended in 1 mL lysis buffer containing 50 mM Tris pH 7.4, 150 mM NaCl, 2 mM EDTA, 0.1% SDS, 0.5% NP-40 and 0.5% deoxycholate. The DNA fragments ranging from 200 to 500 bp were sonicated from suspension, centrifuged at 12,000× *g* for 10 min and collected from the supernatant. For each immunoprecipitation, 300 µL of DNA fragments was incubated with 10 μg PURα antibody (Abcam, Cambridge, MA, USA, Cat#ab125200) and corresponding rabbit anti-Ig G antibody (Cell Signaling Technology, Danvers, MA, USA, Cat#2729P), respectively, overnight at 4 °C, and the immunoprecipitates were further incubated with protein A Dynabeads for 3 h at 4 °C. After applying the magnet and removing the supernatants, the beads were sequentially washed with lysis buffer, high-salt buffer (250 mM Tris pH 7.4, 750 mM NaCl, 10 mM EDTA, 0.1% SDS, 0.5% NP-40 and 0.5 deoxycholate), and PNK buffer (50 mM Tris, 20 mM EGTA and 0.5% NP-40) twice. The immunoprecipitates were eluted from the beads with elution buffer (50 nM Tris pH 8.0, 10 mM EDTA and 1% SDS) and reverse cross-linked by overnight incubation at 65 °C. After sequential RNase A (Thermo, Waltham, MA, USA) and proteinase K (Invitrogen, Carlsbad, CA, USA) treatment, DNA fragments were purified by phenol extraction and ethanol precipitation. To generate libraries for high-throughput sequencing, purified DNA fragments were end-repaired, adenylated, ligated to adaptors and PCR amplified for 18 cycles. The PCR products measuring 250–450 bp were gel-purified, quantified and stored at −80 °C until they were used for sequencing. 

For high-throughput sequencing, the libraries were prepared following the manufacturer's instructions and applied to an Illumina NextSeq 500 (Illumina, San Diego, CA, USA) system for 151-nt paired-end sequencing by ABLife (Wuhan, China). The raw reads obtained after sequencing were processed to obtain the filtered clean reads using FASTX toolkit (http://hannonlab.cshl.edu/fastx_toolkit) to remove low quality (< 20) and short (<16 nt) reads. The quality filtered reads were mapped to the human genome hg38 by Bowtie2 [31]. Only the uniquely mapped reads were kept for further analysis. Enriched binding peaks were generated after filtering through control input by MACS14 (version 1.4) [32] with default thresholds to identify significant PURα binding sites/peaks. Peaks were annotated by using bedtools software (v2.24.0) (https://bedtools.readthedocs.io/en/latest/). Plots showing representative peak regions were generated using Integrated Genome Viewer (version 2.5.2). Motif enrichment analysis was done using HOMER [33] at default parameters. Range of motif widths was set to 4 and 20 as the minimum motif width and maximum motif width, respectively.

ChIP-PCR assay was carried out using the SimpleChIP^®^ Enzymatic Chromatin IP kit (Magnetic Beads) (Cell Signaling Technology, Danvers, MA, USA) by following the manufacturer’s procedure. Briefly, the KYSE170 cells were seeded in 15 cm dishes until they grew to more than 90% of confluence. The full cells were fixed with 1% formaldehyde (16% formaldehyde, methanol-free) (Cell Signaling Technology, Danvers, MA, USA) for 10 min at room temperature (RT), stopped cross-linking with glycine for 5 min at RT, washed twice with ice-cold PBS containing protease inhibitor, added 2 mL ice-cold PBS, scraped off the cells with a scraper and finally centrifuged at 2000× *g* for 5 min at 4 °C to collect cell precipitation. The prepared nuclei were digested into 150–1000 bp chromatin DNA fragments by nuclease and sonication, and then their concentration was determined. Immunoprecipitation was performed with 5 μg PURα antibody (Abcam, Cambridge, MA, USA, Cat#ab125200) and corresponding rabbit anti-Ig G antibody (from the kit), respectively. The immunoprecipitates were incubated with protein G magnetic beads, and the antibody-protein G magnetic bead complex was collected for subsequent elution and reverse cross-linking. Five microliters of initial chromatin DNA fragment lysate were used as an input control. Finally, 2 μL purified DNA was used as template for PCR and qRT-PCR verification. The primer design is shown in Table 2. The final raw data of ChIP-qPCR was also performed by the 2^−∆∆Ct^ calculation method [26].

### 2.4. Western Blot Analysis

Cell lysates were collected by RIPA buffer (CWBIO, Beijing, China) with a protease inhibitor. Then, the lysates were centrifuged at 15,000× *g* for 15 min at 4 °C after sonicating for 30 s on ice. Protein concentration was determined by BCA (Thermo, Waltham, MA, USA) protein assay. Proteins were separated on 10% SDS-PAGE and transferred to a polyvinylidene fluoride (PVDF) membrane. The membrane with protein bands was sealed with 5% skim milk (Becton, Dickinson and Company, Franklin Lakes, NJ, USA) for 1 h at RT, and incubated with diluted antibodies overnight at 4 °C. The membrane was incubated with the corresponding secondary antibody for 1 h at RT after washing with TBST three times. Finally, the membrane was added to chemiluminescence chromogenic solution (Biokits, Barcelona, Spain) after washing 3 times with TBST and was measured by exposure imaging analysis in an ImageQuant LAS4000 chemiluminescence imaging analyser (GE Healthcare Life Sciences, Pittsburgh, PA, USA). The information and dilution multiples of the antibodies are listed in Table 3.

### 2.5. Plasmid Construction

The pCMV6-Myc-DDK-AC control plasmid and pCMV6-PURα (Myc-DDK-tagged) plasmid were purchased from OriGene (Rockville, MD, USA). The pCMV6-PURα-NLS vector was constructed including the coding sequence (CDS) and the C-terminus of the *PURA* gene containing 3× Flag and 3× nuclear localization signal (NLS) sequences (Figure S1).

Construction of pCMV6-ATF4: the CDS regions of the *ATF4* gene (accession numbers: 468) were obtained from NCBI. The corresponding CDS regions of *ATF4* were amplified by employing the whole genome of KYSE170 cells as the template, a 2× GoldStar Best MasterMix (Dye) kit (CWBIO, Beijing, China) and primers. A universal DNA Purification kit (Tiangen, Beijing, China) was used to purify amplified or digested DNA products. In total, 1 μg purified DNA products were double-digested by 1 μL enzyme Xhol and HindIII (New England Biolabs, Ipswich, MA, USA) in 50 μL system at 37 °C. Then, these digested products further were purified and measured. After purification, a Quick Ligation^TM^ kit (New England Biolabs, Ipswich, MA, USA) was used to ligate the fragments with linearized vectors for 5 min at 25 °C. The ligated DNA products were transformed using DH5α competent cells (CWBIO, Beijing, China) and the monoclonal clones were picked out and sequenced.

Construction of PCK2 promoter truncation: the PCK2 promoter region was the sequence between the upstream 2000 bp and downstream 5’ noncoding regions of the transcriptional start site (TSS) of the *PCK2* gene (accession numbers: 5106) from the UCSC Genome Browser website. The three truncated sequences were divided into the regions of 1727 bp (−1500/+227), 1060 bp (−953/+107) and 328 bp (−221/+107) as necessary. Primers were designed at both ends of the truncated regions (Table 4). The corresponding truncated regions of the PCK2 promoter were also amplified by using the whole genome of KYSE170 cells as the template, a 2× GoldStar Best MasterMix (Dye) kit (CWBIO, Beijing, China) and these corresponding truncated primers. Utilizing same reagent and methods above, a pGL3-Basic plasmid (Promega, Wisconsin, WI, USA) and truncated regions of the PCK2 promoter were constructed to the PCK2 promoter truncation.

Construction of the PCK2 promoter S1 site mutant: similarly, the reagent and methods mentioned above were adopted, and pGL3-Basic-PCK2-Luc-1500-227 was employed as the template. A PrimeSTAR^®^ GXL DNA Polymerase kit (Takara, Kyoto, Japan), the purified product amplified by corresponding primers, and a Fasta-II rapid site-directed mutagenesis kit (Sbsgene, Beijing, China) were constructed for the PCK2 promoter S1 site mutant. All primers for the recombinant plasmids are listed in Table 4.

### 2.6. RNAi Interference

KYSE170 cells were seeded in a 6-well plate at a density of 4 × 10^5^ cells/well. Next, cells were transfected with siRNA (400 pmol/well) via Lipofectamine 2000 (Invitrogen, Carlsbad, CA, USA) and Opti-MEM medium (Gibco/BRL, Grand Island, NY, USA) when cells grew for 18-24 h and the convergence degree of cells was 40–50%. After 6 h of transfection, the same volume of RPMI 1640 medium with 20% FBS was added to every well. The sequences of siRNA-PURα (GenePharma, Shanghai, China) are listed in Table 5.

### 2.7. Transfection and Luciferase Assay

Common transfection. KYSE510 and KYSE170 cells were seeded in 6-well plates at a density of 8 × 10^5^ cells/well and 5 × 10^5^ cells/well, respectively. Then, the cells were transfected with plasmid (2 μg cell/well) via Lipofectamine 3000 (Invitrogen, Carlsbad, CA, USA), P3000 (Invitrogen, Carlsbad, CA, USA) and Opti-MEM (Gibco/BRL, Grand Island, NY, USA) medium, when the cells had grown for 18-24 h and the confluence degree of the cells was 60–70%. After 6h of transfection, the same volume of RPMI 1640 medium with 20% FBS was added to every well.

Luciferase assay. KYSE170 cells were seeded in 24-well plates at a density of 9 × 10^4^ cells/well. Next, cells were commonly transfected with RL-TK (100 ng cell/well), pCMV6/pCMV6-PURα (400 ng cell/well) and pGL3-basic-PCK2-luc-truncation/mutant (100 ng cell/well). Fresh medium was placed on cells after 24 h transfection, and the cell lysates were collected after 48 h transfection. The fluorescence intensity was measured by a Dual-Luciferase^®^ Reporter Assay System kit (Promega, Wisconsin, WI, USA) on a Turner chemiluminescence instrument (TurnerBioSystems, Sunnyvale, CA, USA).

### 2.8. Metabolic Phenotypes

Oxygen consumption rate (OCR) and extracellular acidification rate (ECAR) were detected with a Seahorse Bioscience Analyzer (Seahorse XFe96, Agilent, Santa Clara, CA, USA), cell culture medium XF RPMI Base Media (pH 7.4, 500 mL), cell culture plate (Seahorse XFe96 FluxPak mini, Agilent, Santa Clara, CA, USA), Seahorse XF Cell Mito Stress Test kit, Seahorse XF Glycolysis Stress Test kit, XF 1.0 m Glucose Solution (50 mL), XF 100 mM Pyruvate Solution (50 mL) and XF 200 mM Glutamine Solution (50 mL) according instructions. All products and instruments were purchased from Agilent (Santa Clara, CA, USA). On the first day, cells were seeded in a special 96-well plate at a density of 8 × 10^3^ cells/well with 80 μL culture medium per well and cultured overnight for at least 16 h. On the second day, the culture medium was changed to the corresponding ECAR/OCR experimental base medium (ECAR base medium: 2 mM glutamine, pH 7.35; OCR base medium: 10 mM glucose, 2 mM glutamine, 1 mM pyruvate, pH 7.4). For ECAR analysis, cells were detected every 3 min after continuous administration of 10 mM glucose and inhibitors (1 μM oligomycin and 50 mM 2-deoxyglucose). For OCR analysis, cells were detected every 3 min after continuous administration of (2 μM oligomycin, 2 μM MFCCP and 0.5 μM rotenone/antimycin A). A BCA protein assay was employed to standardize the metabolic rates to the number of cells.

### 2.9. Statistical Analysis 

The specific binding peaks of the PURα sample were obtained and calculated by MACS (version 1.4) software [32], taking the input sample as the background. The further screening criteria of differentially expressed genes (DEGs) of ChIP-seq is logFC ≥ 1 or ≤ −1, *p* ≤ 0.01, FDR ≤ 1.5% and that of RNA-seq is logFC ≥ 0.5 or ≤−0.5, *p* ≤ 0.01, FDR ≤ 1.5%. The functions of DEGs were analyzed by Gene Ontology (GO, http://www.geneontology.org/) annotation and gene set enrichment analysis (GSEA, https://www.gsea-msigdb.org/gsea) [34,35]. The KEGG pathway analyses for the DEGs and overlapping genes were performed by KORAS 3.0 [36] (http://kobas.cbi.pku.edu.cn/kobas3/genelist/). Heatmaps for metabolic signatures were created in R using a pheatmap package (https://cran.r-project.org/web/packages/pheatmap/index.html). Peak visualizations of ChIP-seq for candidate genes were analyzed and magnified by IGV software [37,38].

The experimental data were analyzed and plotted by GraphPad Prism 8.0 (San Diego, CA, USA) and calculated as means ± SD. Significant differences between the experimental group and the control group were determined by Student’s t test. The figures are shown as follows: *** *p* < 0.001, ** *p* < 0.01, * *p* < 0.05, ns *p* > 0.05. *P*-values < 0.05 were considered to be significant.

## 3. Results

### 3.1. Motif and Function Analysis of ChIP-seq

**Figure 1:**
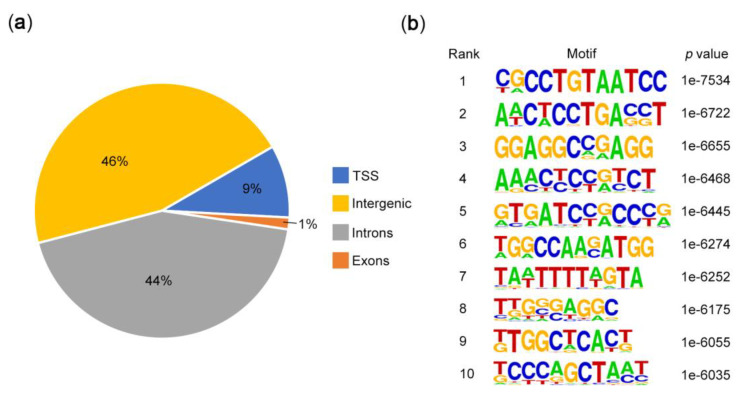
Peak and motif analysis of ChIP-seq. (**a**) A pie chart of peak distribution on gene elements. (**b**) Motif scan analysis of significantly enriched DNA-binding regions not collected in the Homer database.

As a multifunctional RNA and DNA binding protein, PURα not only regulates DNA replication and transcription but also plays an important role in binding with RNA [39,40,41]. To investigate the features of PURα binding motifs, we performed a ChIP-seq analysis in KYSE510 cells, which yielded a total of 56974 peaks and 2353 peak-associated genes were detected. Among the total peaks, a marked enrichment for introns and exons is shown in a pie chart (Figure 1a). Next, we identified the top 10 motif elements of PURα through motif scan analysis (Figure 1b), which indicated that PURα can bind to several new motifs, such as “TA”, “TC” and “TTN”, apart from the reported “GA”, “GC” and “GGN” [42,43,44].

**Figure 2:**
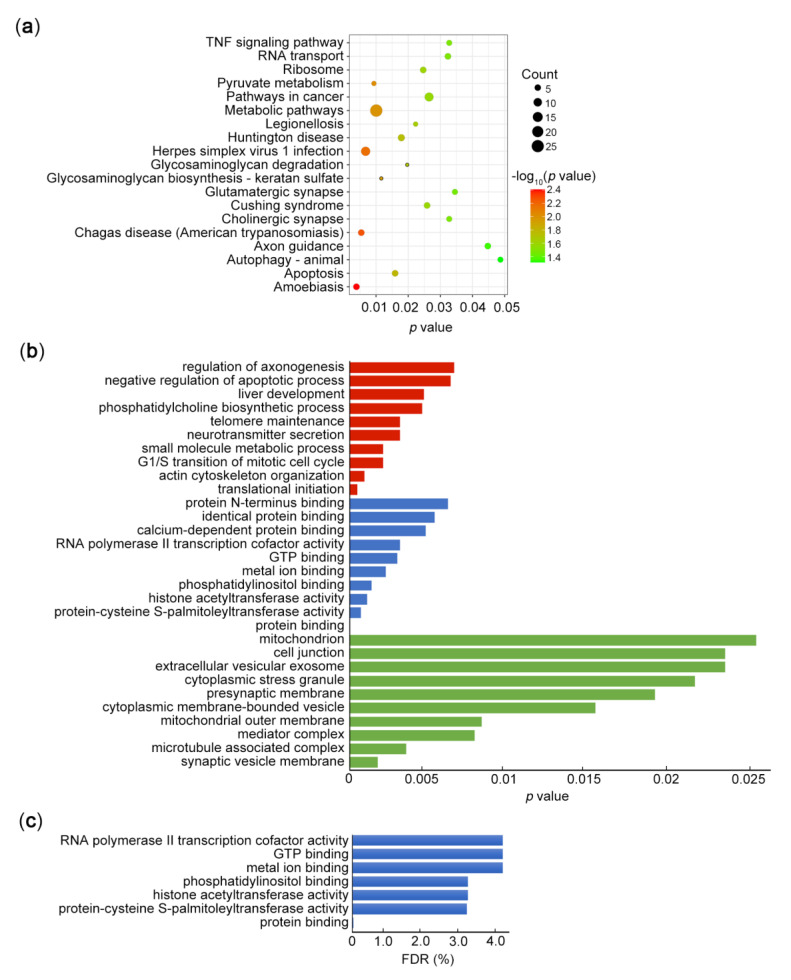
KEGG and GO analysis of ChIP-seq. (**a**) The significantly enriched KEGG pathways are shown based on ChIP-seq data in KYSE510 cells after PURα overexpression (*p* < 0.05). (**b**) The top 10 enriched biological process (BP), cellular component (CC) and molecular function (MF) of the same ChIP-seq data via GO analysis (*p* < 0.05, the red, blue and green bars refer to CC, BP and MF, respectively). (**c**) GO analysis of ChIP-seq via FDR correction shown the top 7 enriched BP functions (*p* < 0.05, FDR < 5%).

Moreover, GO analyses were performed to investigate the biological processes and functions associated with PURα. Interestingly, KEGG pathway analysis showed a marked enrichment of metabolic pathways, herpes simplex virus 1 infection and amoebiasis (Figure 2a). The top 10 enriched biological process (BP), cellular component (CC) and molecular function (MF) terms are listed in Figure 2b. The GO analysis demonstrated that PURα was correlated with translational initiation, protein binding and synaptic vesicle membrane, implying multiple functions of PURα in biological processes. We further carried out FDR correction for the pathway and functional results enriched by KEGG and GO analysis, respectively. In the KEGG analysis, we found that the FDR values of the pathways with *p* < 0.05 were close to 1 (not shown), suggesting that these enrichment pathways were not significant. Similarly, among the GO function enrichment of BP, CC and MF, except for BP type, the other two types have higher FDR values (Figure 2c). These results corrected by FDR may more strictly reflect the gene functions of PURα binding.

### 3.2. Screening of Potential Candidate Regulated Genes for PURα

**Figure 3:**
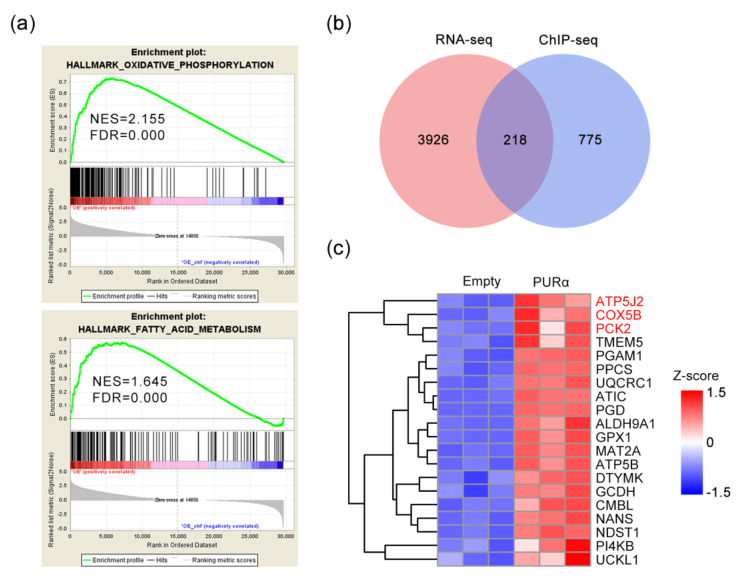
Analysis of RNA-seq and ChIP-seq. (**a**) GSEA of PURα-regulated genes with normalized enrichment score (NES) and false-discovery rate (FDR). (**b**) Venn diagram showing the overlap of DEGs between RNA-seq (logFC ≥ 0.5 or ≤ −0.5, *p* ≤ 0.01, FDR ≤ 1.5%) and ChIP-seq (logFC ≥ 1 or ≤ −1, *p* ≤ 0.01, FDR ≤ 1.5%). (**c**) Heat map depicting changes in gene expression levels involved in fatty acid degradation, adipocytokine signalling pathway, oxidative phosphorylation and glycolysis/gluconeogenesis based on the overlapping genes. The raw sequence data reported in this paper have been deposited in the Genome Sequence Archive in National Genomics Data Center, Beijing Institute of Genomics (China National Center for Bioinformation), Chinese Academy of Sciences, under accession number CRA003437 that are publicly accessible at https://bigd.big.ac.cn/gsa.

Previous research has determined that PURα promotes the invasion and migration of ESCC cells via epithelial-mesenchymal transition (EMT) [9]. To investigate other functions of PURα in ESCC cells, we analyzed the RNA-seq data via GSEA. As expected, a significant increase in metabolic pathways, including oxidative phosphorylation and fatty acid metabolism genes, was observed after PURα overexpression (Figure 3a). These results suggest that PURα may play an important role in metabolism. 

To further explore whether PURα regulates metabolic genes, we analyzed RNA-seq and ChIP-seq data to identify optimal candidates. First, we evaluated the overlap of the pre-processed DEGs between RNA-seq and ChIP-seq, as shown in the Venn diagram. There were 218 overlapping genes (Figure 3b). Next, gene enrichment analysis demonstrated a significant increase in metabolic pathways, including fatty acid degradation, adipocytokine signaling pathway, oxidative phosphorylation and glycolysis/gluconeogenesis genes, upon PURα overexpression (Figure 3c). The top 3 genes were *ATP5J2*, *COX5B* and *PCK2*.

### 3.3. Transcriptional Activation of thePCK2 Promoter Depends on the GGN Motif bound by PURα

**Figure 4:**
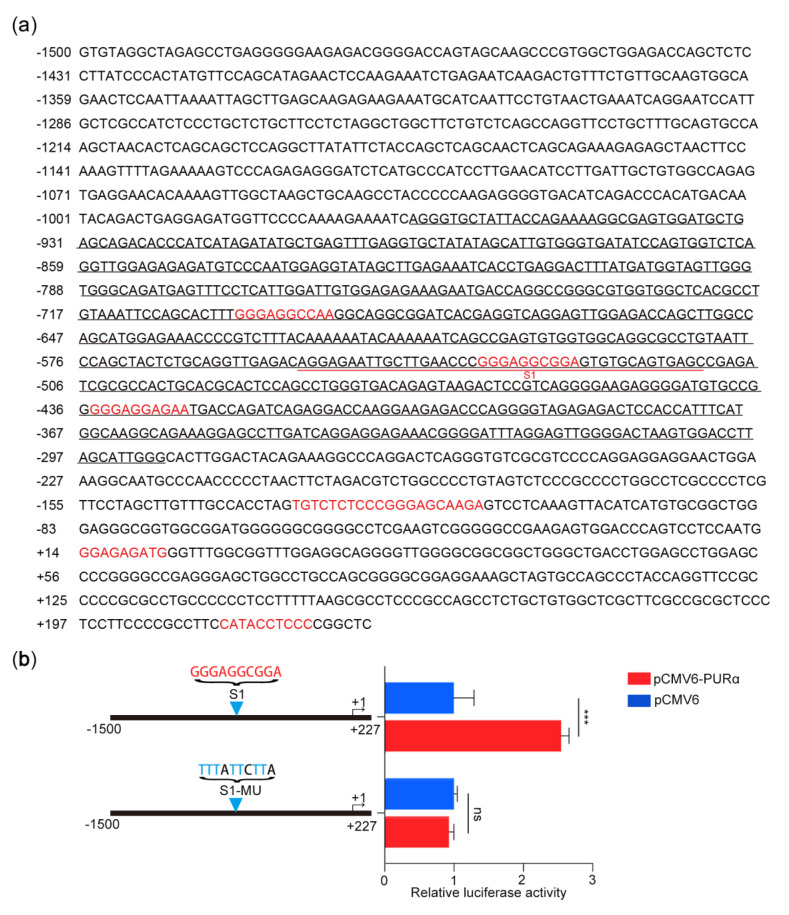
Specific motif of PURα binding to the PCK2 promoter region. (**a**) Online prediction of the PURα binding sites in the PCK2 promoter region (−1500/+227). (**b**) Luciferase assay of the point mutant constructor at prediction site S1 in KYSE170 cells under exogenous overexpression of pCMV6/pCMV6-PURα. *n* = 3. ***, *p* < 0.001; ns, *p* > 0.05.

**Figure 5:**
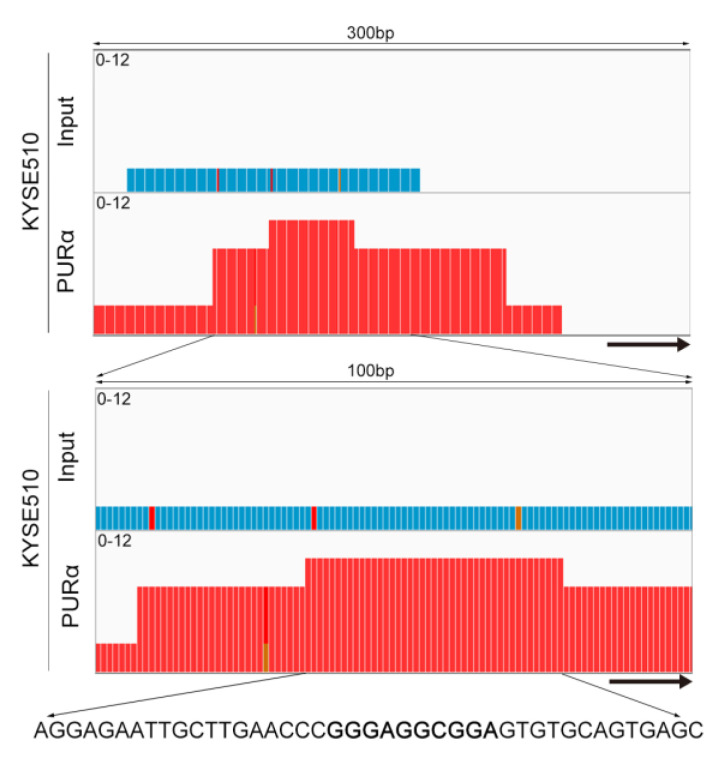
IGV visual analysis. Visual analysis of the PURα binding peak in the PCK2 promoter region from ChIP-seq of KYSE510-input/PURα cells by IGV. The arrows refer to the *PCK2* gene.

Considering that PCK2 plays an important rate-limiting role in gluconeogenesis and participates in the synthesis of glycerol, amino acids and nucleotides and cataplerosis in the tricarboxylic acid (TCA) cycle [45,46], we focused on the regulation of PCK2 by PURα. To investigate whether PURα binds to the promoter region of the *PCK2* gene, we downloaded the sequence of the PCK2 promoter region (−1500/+227) from the UCSC Genome Browser and predicted whether there were PURα binding sites in the promoter region of *PCK2* gene through ALGGEN-PROMO [47]. Indeed, there were seven predicted binding sites in this region (Figure 4a). To further determine which predicted binding sites of PURα may play a role, the PURα binding peak in the promoter region of the *PCK2* gene from ChIP-seq was magnified and analysed by IGV. There were three predicted binding sites of PURα in the binding peak region (Chr14:24093172-24093825), which were GGGAGGCCAA, GGGAGGCGGA and GGGAGGAGAA, respectively (Figure 4a). To elucidate the exact binding sequence, we enlarged the visual peak region and obtained the specific sequence of the highest peak, which was AGGAGAATTGCTTGAACCCGGGAGGCGGAGTGTGCAGTGAGC (Figure 5). In addition, this specific sequence contained the S1 (GGGAGGCGGA) motif. These results suggested that PURα may be involved in the transcriptional regulation of the *PCK2* gene.

To better determine whether the S1 motif is a specific binding site for PURα, we designed the mutant S1-MU as a luciferase reporter construct for verification. Under the condition of exogenous overexpression of pCMV6/pCMV6-PURα, we measured the luciferase activity of the mutant S1-MU construct in KYSE170 cells. Compared with the control group, the S1-MU group had almost no activity, indicating that PURα binds to the GGGAGGCGGA motif (-534, -525) of the PCK2 promoter (Figure 4b).

**Figure 6:**
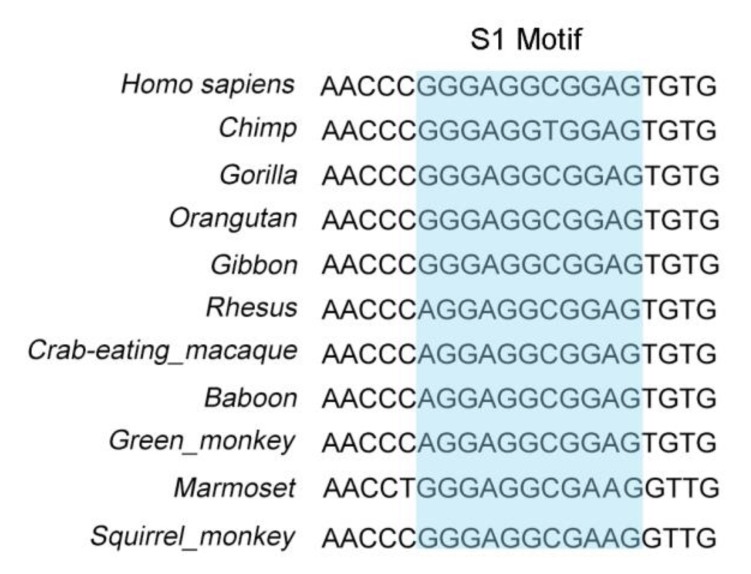
Sequence analysis of the S1 motif in the PCK2 promoter region of different primates.

Next, to confirm the conservation of the regulation of the transcriptional activation of PCK2 by PURα, we compared and analyzed the S1 motif of the PCK2 promoter in different primates. We found that compared with the S1 locus upstream of the human *PCK2* gene, most primates had the same S1 locus, while the minority had some differences in the sequence, and the overall motif was highly similar (Figure 6). This result indicates that the transcriptional regulation of PCK2 by PURα may be ubiquitous in primates.

### 3.4. Identification of the Core Binding Region of the PCK2 Gene for PURα

**Figure 7:**
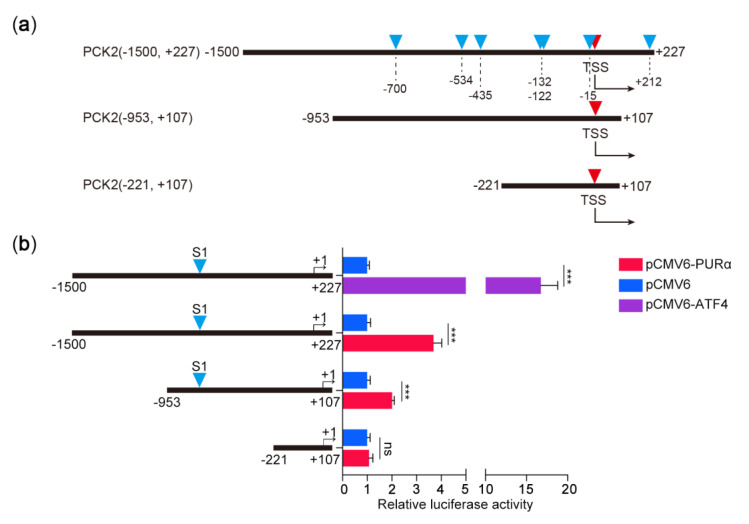
Identification of PURα binding to the core promoter region of PCK2. (**a**) Schematic diagram showing the truncated region of the PCK2 promoter and 7 predicted PURα binding sites. The red triangle represents the TSS, and the blue triangle represents PURα predicted binding sites. (**b**) Luciferase assay of the positive control and truncated PCK2 promoter in KYSE170 cells under the condition of exogenous overexpression of pCMV6/pCMV6-PURα. *n* = 3. ***, *p* < 0.001; ns, *p* > 0.05.

To examine the core binding region of the *PCK2* gene for PURα, the PCK2 promoter region was truncated into three fragments (−1500/+227; 953/+107; and −221/+107). These fragments were engineered as truncated luciferase reporter constructs (Figure 7a). Under the condition of exogenous overexpression of pCMV6/pCMV6-PURα, we detected luciferase activity after transient transfection of these truncated constructs into KYSE170 cells. Additionally, we engineered a pCMV6-ATF4 [24] construct as a positive control for the luciferase assay. Clearly, compared with the pCMV6 group, the positive control and negative PCK2 (−221/+107) groups displayed almost 16-fold higher and no relative luciferase activity, respectively (Figure 7b). Although the relative luciferase activity of PCK2 (−1500/+227) was considerably higher than that of PCK2 (−953/+107) under the condition of exogenous overexpression of PURα, the overall trend of luciferase activity was the same (Figure 7b). These results demonstrated that PURα may bind to the upstream region (−953/−221) of TSS in the *PCK2* gene.

**Figure 8:**
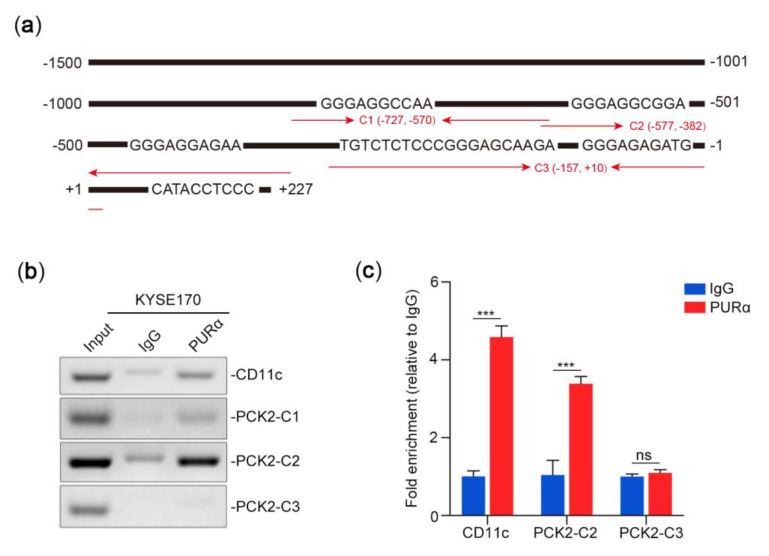
PURα binds directly to the promoter region of PCK2. (**a**) Schematic diagram showing the specific motifs of 7 PURα predicted binding sites and the designed C region verified by ChIP-PCR (C1: −727/−570; C2: −577/−382; C3: −157/+10). (**b**) ChIP-PCR analysis of PURα binding to the PCK2 promoter region (C1, C2 and C3) and CD11c as a positive control in KYSE170 cells. (**c**) ChIP-qPCR analysis of PURα binding to the PCK2 promoter C2/C3 region and CD11c in KYSE170 cells. *n* = 3. ***, *p* < 0.001; ns, *p* > 0.05.

Based on PURα binding to the S1 motif of the upstream region (-953/-221) of TSS in the *PCK2* gene, we further examined whether PURα binds directly to this motif. According to the predicted sites and truncation experiments, we selected C1, C2 and C3 regions for ChIP-PCR/qPCR verification. As shown in Figure 8a, PURα can not only directly bind to the CD11c promoter as a positive control [48] but can also bind to the C1 and C2 regions of the PCK2 promoter. Since the binding band between PURα and the C2 region was stronger than that between PURα and C1, we selected the C2 region and the CD11c promoter for further qPCR verification (Figure 8b,c). The luciferase assay of truncated constructs and the ChIP-PCR/qPCR results further confirmed that the core promoter region of the *PCK2* gene is the upstream -727/-382 region of TSS.

### 3.5. PURα Activates the Transcription and Expression of PCK2

**Figure 9:**
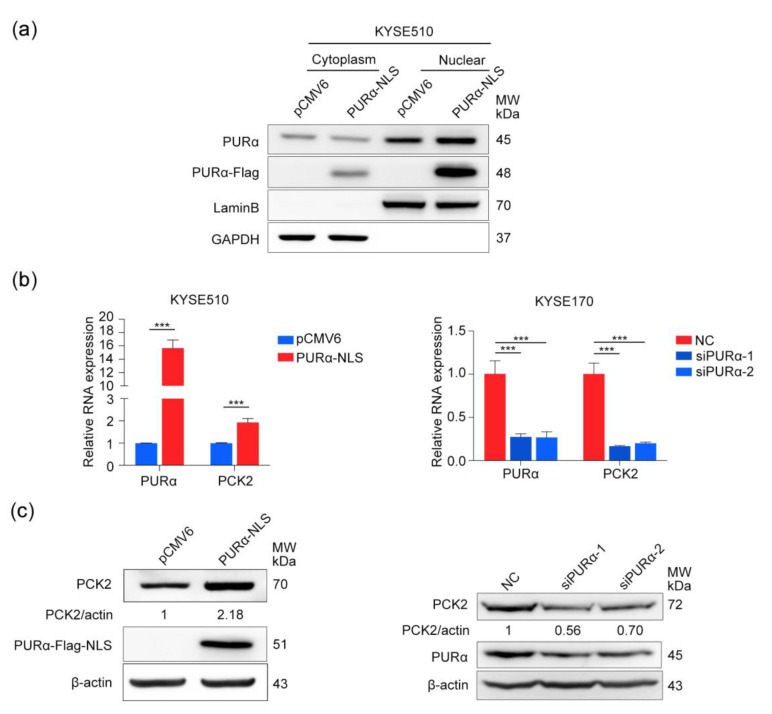
PURα activates the transcription and expression of PCK2. (**a**) The cytoplasmic/nuclear protein level of PURα in the KYSE510 cells of overexpressing pCMV6-PURα-NLS. (**b**) The mRNA level of PCK2 in the KYSE510 cells of overexpressing pCMV6-PURα-NLS or KYSE170 cells with PURα knockdown was detected by qPCR. (**c**) The protein level of PCK2 in the KYSE510 cells of overexpressing pCMV6-PURα-NLS or the KYSE170 cells with PURα knockdown was verified by Western blotting. *n* = 3. ***, *p* < 0.001.

To determine whether the transcriptional activation of PCK2 by PURα can affect the expression and enzyme activity of PCK2, qRT-PCR and Western blot analyses were performed. Overexpression of the common pCMV6-PURα vector did not change the protein and mRNA levels of PCK2 in KYSE510 cells (Figure S2). Because PURα is a cytoplasmic/nuclear protein, considering that PURα acts as a transcription factor in the nucleus, we reconstructed another PURα vector only expressed in the nucleus, pCMV6-PURα-NLS. After the pCMV6-PURα-NLS vector was overexpressed in KYSE510 cells, PURα was determined to be overexpressed in the nucleus compared with the cytoplasm (Figure 9a). First, the mRNA levels of PCK2 in KYSE510 cells overexpressing pCMV6-PURα-NLS and KYSE170 cells knocked down for PURα were detected by qRT-PCR, and it was found that PURα could indeed promote the transcriptional activation of PCK2 (Figure 9b). Next, through Western blot analysis overexpression of PURα in KYSE510 cells could also promote the expression of PCK2. Similarly, the expression level of PCK2 decreased after knockdown of PURα in KYSE170 cells (Figure 9c). These findings confirmed that PURα can not only activate the transcription of PCK2 but can also promote its expression. 

### 3.6. PURα Promotes the Mitochondrial Respiration and Glycolysis in ECSCs

**Figure 10:**
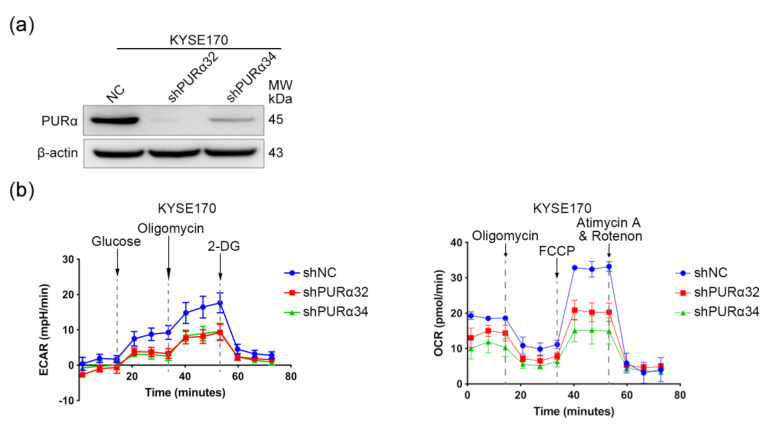
PURα promotes mitochondrial respiration and glycolysis in ESCC cells. (**a**) The protein levels of PURα in KYSE170-shNC/shPURα32/34 cells were assessed by Western blotting. (**b**) Oxygen consumption rate (OCR) and extracellular acidification rate (ECAR) detection in KYSE170-shNC/shPURα32/34 cells.

To investigate whether the ESCC cells affected by PURα have metabolic phenotypic changes, KYSE170-shPURα32/34 cells were used to detect OCR and ECAR. As expected, when glucose was added, the ECAR in the KYSE170-shPURα32/34 groups was significantly lower than those in the control group, implying that knocking down PURα can reduce the basal glycolysis and maximum glycolytic capacity of KYSE170 cells (Figure 10b). Similarly, when FCCP, an uncoupling agent, was added to medium, the OCR in the KYSE170-shPURα32/34 groups was significantly lower than those in the control group, implying that knocking down PURα can reduce the maximal mitochondrial respiratory and spare respiratory capacity of KYSE170 cells (Figure 10b). Summarily, the results indicate that knocking down PURα can significantly reduce the mitochondrial respiration and glycolysis ability of KYSE170 cells.

## 4. Discussion

It has been observed that both PURα and heterogeneous nuclear ribonucleoprotein K (hnRNP-K) bind to the *CD43* gene promoter and co-inhibit its transcriptional activity in the process of leukocyte differentiation [49,50]. In addition, PURα can also bind to the CD11cβ2 integrin gene promoter to induce its transcription and expression [48]. Furthermore, PURα promotes myogenesis by downregulating MHC transcription [8] and attenuates the transactivation of the smooth muscle actin gene in myofibroblasts [51]. These studies indicate that PURα plays a vital role in transcriptional regulation. A patient with a frame-shift deletion in the *PURA* gene not only has the classical PURα deficiency phenotype, but also has a significant hypoglycorrhachia. This phenotype overlaps with the clinical manifestations of glucose transporter type 1 (GLUT1) deficiency syndrome, suggesting that PURα may be involved in the regulation of GLUT1 or affect the function of glucose metabolism [52]. However, the relationship and mechanism between PURα and tumor metabolism have not been elucidated, and research on the regulation of metabolic genes by PURα has not been reported. Therefore, we first performed ChIP-seq analysis of KYSE510-input/PURα cells to identify the specific binding motifs, candidate genes and enriched functions of PURα. Next, we analyzed RNA-seq data via GSEA to determine the relationship between PURα and metabolism. Then, we validated the optimal candidate genes related to metabolism from the overlap of between RNA-seq and ChIP-seq data. We observed a metabolic gene, *PCK2*. Further IGV visual analysis showed that there was a PURα binding peak in the PCK2 promoter.

To investigate the transcriptional regulation of PCK2 by PURα, we first predicted the binding sites in the PCK2 promoter bound by PURα via ALGGEN-PROMO and UCSC. Then, mutant constructs of the PCK2 promoter were designed through predicted sites. Finally, the specific site S1 (GGGAGGCGGA) of the PURα binding PCK2 promoter was confirmed by luciferase assay. Moreover, ChIP-PCR/qPCR and the luciferase assay of the truncated PCK2 promoter that PURα directly bound to the core promoter region (−727/−382) of *PCK2*. PURα is a highly conserved protein, therefore, it is speculated that the transcriptional regulation of the *PCK2* gene by PURα is conserved. To investigate this possibility the S1 motif of the PCK2 promoter in different primates was analyzed by searching the UCSC and NCBI databases. It was found that all the motifs of different species were highly similar, suggesting that the transcriptional regulation of PCK2 by PURα may be common in other primates.

In recent years, PCK2 has been abnormally expressed in a variety of cancers, such as liver cancer, colorectal cancer, lung cancer, melanoma and prostate cancer, which is closely related to the occurrence and development of cancer [53,54,55,56,57]. Sequence analysis of the PCK2 promoter region revealed that it contained several potential regulatory elements, including the SREBP, CREB, C/EBP, AP-1, AP-2 and SRY elements [58]. However, Stark et al. believed that PCK2 is a weaker molecule for rapid transcriptional regulation because of its mitochondrial location [59]. In contrast, cyclic AMP-dependent transcription factor (ATF4) enhances the transcriptional activity of PCK2 and activates its dependent pro-survival pathways under amino acid deprivation or endoplasmic reticulum stress by binding to the ATF/CRE site in the PCK2 promoter. PCK2 participates in the supportive adaptations of breast and cervical cancer cells to adapt to the stress state in the tumor environment [60]. In addition, hypoxia-inducible factor 1-α (HIF1-α) in breast cancer negatively regulates PCK2 at the transcriptional level causing breast cancer tumor-repopulating cells (TRCs) to grow in anoxic environments [61]. Therefore, although PCK2 is located in the mitochondria, it is still subject to rapid transcriptional regulation and subsequently participates in the process of tumorigenesis and development.

Since PURα can activate the transcription of PCK2, we hypothesized that PCK2 must perform its metabolic enzyme function in the form of protein. Therefore, the question arose of whether the transcriptional activation of PCK2 by PURα further affects its expression. Western blot analysis showed that PURα not only activated the transcription of PCK2 but also promoted its expression. Previous research has indicated that PURα promotes the invasion and migration of ESCC cells via EMT [9]. Additionally, EMT and metabolic reprogramming in various cancer cells is related. EMT-related transcription factors can not only promote EMT but also regulate metabolic genes in metabolic pathways. Conversely, crucial metabolic enzymes involved in metabolic reprogramming, such as glycolysis, mitochondrial metabolism, lipid metabolism and glutaminolysis, also activate EMT in cancer cells [62]. Overall, these findings indicate that PURα promotes EMT and the transcriptional activation of the *PCK2* gene in ESCC cells, while PCK2 can participate in the metabolic reprogramming of cancer cells. Therefore, we hypothesized that PURα affects metabolic reprogramming and EMT in ESCC cells through transcriptional activation of PCK2. OCR and ECAR detection were employed to investigate the metabolic phenotypic changes of ESCC cells affected by PURα. Indeed, PURα promotes the mitochondrial respiration and glycolysis in ESCC.

In general, this study identified other new binding motifs and functions of PURα in ESCC cells. Meanwhile, our findings suggest that PURα regulates the transcription and expression of the metabolic enzyme PCK2 and the metabolism of ESCC cells. However, whether PURα can affect the metabolism and EMT of ESCC cells by the transcriptional regulation of PCK2 is not known and warrants further research.

## 5. Conclusions

In the present study, we discovered novel binding motifs of PURα on a genome-wide scale and its other functions, including metabolic pathway, translational initiation and protein binding. We identified the regulatory mechanism by which PURα activates the transcription and expression of PCK2 in ESCC cells. In addition, PURα can promote mitochondrial respiration and glycolysis in ESCC. Based on the stimulatory effect of PURα on EMT and the important role played by PCK2 in tumor metabolism, PURα and PCK2 may represent new therapeutic targets for ESCC.

## Figures and Tables

**Table 1:** Primers of the target gene.

Primer Name	Sequence (5’→3’)
Human *PCK2*-F	GGGCACTACCTGGAACACTG
Human *PCK2*-R	GCACTGTCCTCCCCCT-CTAA
Human *PURA*-F	TACGGCGTGTTTATGCGAGT
Human *PURA*-R	TTGCAGAAGGTGTGTCCGAA
Human *β-actin*-F	CATGTACGTTGCTATCCAGGC
Human *β-actin*-R	CTCCTTAATGTCAC-GCACGAT

**Table 2:** ChIP-PCR/qPCR primers.

Primer Name	Sequence (5’→3’)
PCK2-C1-F	GCTCACGCCTGTAAATTCCAG
PCK2-C1-R	TAGCTGGAATTACAGGCGC
PCK2-C2-F	TCCAGCTACTCTGCAGGTT
PCK2-C2-R	TCTCTCTACCCCTGGGTCT
PCK2-C3-F	CGTTCCTAGCTTGTTTGCCAC
PCK2-C3-R	CCAAACCGCCAAACCCATCT
CD11c-F	TCCATCTAAGCAAAGGGCATCA
CD11c-R	GCCAGGGGAAGGAAGAAGATT

**Table 3:** Information and dilution multiple of antibodies.

Antibody	Source	Identifier	Dilution Multiple
PCK2-Rabbit antibody	Cell Signaling Technology	Cat# 6924S	1:1000
PURA-Rabbit antibody	Abcam	Cat# ab79936	1:1000
β-actin-Mouse antibody	Boao rui jing	Cat# ab1015t	1:5000

**Table 4:** Primers for the recombinant plasmids.

Primer Name	Sequence (5’→3’)
*PCK2*-Luc-1500-227-F	CCGCTCGAGGTGTAGGCTAGAGCCTGAG
*PCK2*-Luc-1500-227-R	CCGCTCGAGGTGTAGGCTAGAGCCTGAG
*PCK2*-Luc-953-107-F	CCGCTCGAGCAGAAAAGGCGAGTGGATGC
*PCK2*-Luc-953-107-R	CCCAAGCTTTGGCACTAGCTTTCCTCCG
*PCK2*-Luc-221-107-F	CCGCTCGAGATGCCCAACCCCCTAACTTC
*PCK2*-Luc-221-107-R	CCCAAGCTTTGGCACTAGCTTTCCTCCG
*PCK2*-Luc-S1-MU-F	CCCTTTATTCTTAGTGTGCAGTGAGCCGAGATCG
*PCK2*-Luc-S1-MU-R	GCACACTAAGAATAAAGGGTTCAAGCAATTCTCCTGTCTCA
pCMV6-*ATF4*-F	CCCAAGCTTATGACCGAAATGAGCTTCCT
pCMV6-*ATF4*-R	CCGCTCGAGGGGGACCCTTTTCTTCCC

**Table 5:** siRNA/shRNA sequences of PURα.

siRNA	Sequence (5’→3’)
siCtrl	UUCUCCGAACGUGUCACGU
siPURα-1	CCACCUAUCGCAACUCCAUTT
siPURα-2	CCAAGUUCGGACACACCUUTT
shNC	ACAGAAGCGATTGTTGATC
shPURα32	CCACCAACTGACAGTTTCTCT
shPURα34	AGCCGCCTTACTCTCTCCATG

## Supplementary Materials

The following are available online at https://www.mdpi.com/2073-4425/11/11/1301/s1, Figure S1: The sequence of the pCMV6-PURα-NLS plasmid. Figure S2: The mRNA/protein level of PCK2 in KYSE510 cells of overexpressing pCMV6-PURα was not changed.
