# Supplementary material for: PURα Promotes the Transcriptional Activation of PCK2 in Esophageal Squamous Cell Carcinoma Cells
Source: Genes (Basel). 2020 Oct 31;11(11):1301. doi: 10.3390/genes11111301 (PMC7692967; doi:10.3390/genes11111301)
Supplement: Supplementary file 1 [file genes-11-01301-s001.pdf]

genes

MDPI

PURA(NM_005859.3)-3×Flag-3×NLS

ATGGCGGACCGAGACAGCGGCAGCGAGCAGGGTGGTGCGGCGCTGGGTTCGGGCGGCTCCCTGG
GGCACCCCGGCTCGGGCTCAGGCTCCGGCGGGGGCGGTGGTGGCGGCGGGGGCGGCGGCGGCA
GTGGCGGCGGCGGTGGCGGGGCCCCAGGGGGGCTGCAGCACGAGACGCAGGAGCTGGCCTCCAA
GCGGGTGGACATCCAGAACAAGCGCTTCTACCTGGACGTGAAGCAGAACGCCAAGGGCCGCTTCCT
GAAGATCGCCGAGGTGGGCGCGGGCGGCAACAAGAGCCGCCTTACTCTCTCCATGTCAGTGGCCGT
GGAGTTCCGCGACTACCTGGGCGACTTCATCGAGCACTACGCGCAGCTGGGCCCCAGCCAGCCGCC
GGACCTGGCCCAGGCGCAGGACGAGCCGCGCCGGGCGCTCAAAAGCGAGTTCCTGGTGCGCGAGA
ACCGCAAGTACTACATGGATCTCAAGGAGAACCAGCGCGGCCGCTTCCTGCGCATCCGCCAGACGG
TCAACCGGGGGCCTGGCCTGGGCTCCACGCAGGGCCAGACCATTGCGCTGCCCGCGCAGGGGCTC
ATCGAGTTCCGTGACGCTCTGGCCAAGCTCATCGACGACTACGGAGTGGAGGAGGAGCCGGCCGAG
CTGCCCGAGGGCACCTCCTTGACTGTGGACAACAAGCGCTTCTTCTTCGATGTGGGCTCCAACAAGT
ACGGCGTGTTTATGCGAGTGAGCGAGGTGAAGCCCACCTATCGCAACTCCATCACCGTGCCCTACAA
GGTGTGGGCCAAGTTCGGACACACCTTCTGCAAGTACTCGGAGGAGATGAAGAAGATTCAAGAGAAG
CAGAGGGAGAAGCGGGCTGCCTGTGAGCAGCTTCACCAGCAGCAACAGCAGCAGCAGGAGGAGACC
GCCGCTGCCACCCTGCTACTGCAGGGTGAGGAAGAAGGGGAAGAAGATGACTACAAAGACCATGACG
GTGATTATAAAGATCATGACATCGACTACAAGGATGACGATGACAAGGATCCAAAAAAGAAGAGAAAGG
3 × Flag
TAGATCCAAAAAAGAAGAGAAAGGTAGATCCAAAAAAGAAGAGAAAGGTATGA
3 × NLS

Figure S1. Sequence of the pCMV6-PURα-NLS plasmid. The cDNA encoding the human *PURA* gene (NM_005859.3) and C-terminal containing 3 × Flag and 3 × NLS sequences were subcloned into the pCMV6-Myc-DDK-AC vector to generate the recombinant vector pCMV6-PURα-NLS. NLS, nuclear localization signal.

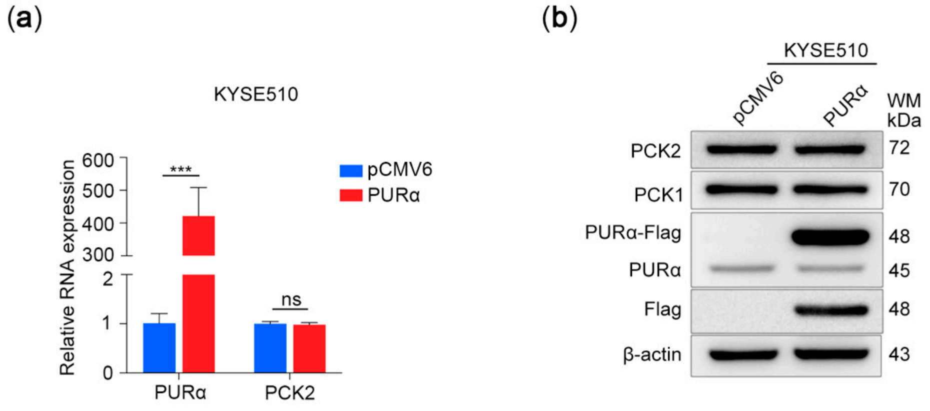

Figure S2. mRNA/protein level of PCK2 in KYSE510 cells overexpressing pCMV6-PURα was not changed. (a) and (b) The mRNA/protein level of PURα in KYSE510 cells was significantly increased by overexpressing pCMV6-PURα, but those of PCK2 were not changed compared with their corresponding control. ***, $p < 0.001$; ns, $p > 0.05$.
